# Natural Bioactive Compounds in Organic and Conventional Fermented Food

**DOI:** 10.3390/molecules27134084

**Published:** 2022-06-24

**Authors:** Barbara Breza-Boruta, Anna Ligocka, Justyna Bauza-Kaszewska

**Affiliations:** Department of Microbiology and Food Technology, Faculty of Agriculture and Biotechnology, Bydgoszcz University of Science and Technology, 6 Bernardynska St., 85-029 Bydgoszcz, Poland; anna.ligocka@pbs.edu.pl (A.L.); justyna.bauza-kaszewska@pbs.edu.pl (J.B.-K.)

**Keywords:** lactic acid bacteria β-carotene, vitamin C, bacteriocins, calcium, fermented food conventional food, organic food

## Abstract

Compared to conventional agriculture, organic farming is believed to provide a higher nutritional and health value in its products due to the elimination of harmful contaminants (pesticides, nitrates, heavy metals, etc.). Numerous studies have been conducted to show how the production system affects the quality of food in terms of the content of bioactive compounds. The aim of this study was to compare the content of some bioactive compounds (vitamin C, β-carotene, Ca content) and lactic acid bacteria (LAB) number and their bacteriocinogenic activity in organic and conventional fermented food. Although the results do not provide an unambiguous conclusion regarding the superiority of one production system over the other, the LAB number in organic pickled carrot juice, sauerkraut, yogurt, and kefir was higher than in their conventional counterparts. Their bacteriocinogenic potential against selected pathogens was also higher in most organic products. Organic vegetables contained significantly more vitamin C, and the calcium content in the organic yogurt was higher compared to the conventional version of the product. Relatively similar concentrations of ß-carotene for both production systems were found in carrot juice, while in organic pickled beet juice, there was five-fold less ß-carotene than in conventional juice.

## 1. Introduction

Conventional agricultural production methods are aimed to increase crop yields and breeding efficiency while reducing food production costs. This goal can be achieved through the use of artificial fertilizers, pesticides, and growth promoters, which, however, reduce the quality of food products. Conscious consumers increasingly seek food from organic farming, where the production process is safe for the environment and human health. The organic production system allows for obtaining raw materials and food containing more bioactive ingredients and significantly less or no nitrates, nitrites, and pesticide residues than crops from conventional farming [1,2]. Organic products usually have a higher vitamin content, especially vitamin C and B vitamins–this mainly applies to potatoes, vegetables, milk, and cereal products. As concluded by Rembiałkowska et al. [3,4], organic potatoes can have an important anti-carcinogenic impact on the human organism due to their lower level of nitrates and simultaneously higher content of vitamin C and phenolic compounds. Organic products also have an increased content of minerals. More iron, magnesium, phosphorus, and potassium were found in cherries, carrots, potatoes, savoy cabbage, spinach, leeks, and lettuce, while an increased amount of calcium was reported in milk [5]. 

Despite many reports confirming the better quality of organic products, many authors indicate no significant differences or even better quality of conventional products. For example, crops treated with mixed fertilizers (organic and chemical) were abundant in β-carotene and minerals but contained lower concentrations of B vitamins than crops grown organically [6]. Ismail and Fun [7] have determined the contents of riboflavin, β-carotene, and vitamin C in some vegetables and found that only a few of them grown organically had a higher content of these compounds. Likewise, lettuce and soybean seeds from conventional systems contained more Ca, Mg, Mn, Fe, Cu P, K, Cu, and Ni than those from organic production [8,9], whereas there were no differences in the mineral content of wheat, barley, faba, bean, and potato [10]. 

In the processing of food produced both ecologically and conventionally, lactic acid fermentation (LAF) is widely used, performed by bacteria from the *Lactobacillus*, *Streptococcus*, or *Lactococcus* genera, which are also naturally present in the human body [11]. This process allows to obtain products with high sensory and nutritional values and to extend the shelf life of food. During fermentation, the concentration of many bioactive compounds increases, and the bioavailability of iron, vitamin C, β-carotene, or betaine is also improved. According to Sangija et al. [12], lactic acid fermentation increased the contents of β-carotene and minerals but reduced vitamin C, total phenols, and chlorophyll levels. Among the lactic acid bacteria (LAB), many strains with probiotic properties are present in fermented products. Their pro-health function consists of inhibiting the development of pathogens in the digestive tract through metabolic products such as bacteriocins, organic acids, diacetyl, hydrogen peroxide, and carbon dioxide, or by strengthening the immunity [13,14]. The most popular fermented food includes dairy products (e.g., kefir, yogurt, buttermilk, cheese) and plant-based products (pickled vegetables, fermented juices). The demand for the latter is shaped by the trend of vegetarianism and the increasing prevalence of lactose intolerance. Certainly, fermented foods based on organic raw materials should be among the products with health-promoting properties that contain a wide range of bioactive compounds. The combination of the lactic acid fermentation process with organic farming products gives hope to obtaining health-promoting food of high quality.

The aim of this study was to determine the number and bacteriocinogenic properties of lactic acid bacteria and to analyze the content of selected bioactive ingredients in fermented organic and conventional products. The LAB antagonistic properties were assessed against selected bacteria representing a wide range of pathogens that can contaminate food products.

## 2. Results

The lactic acid bacteria (LAB) number and the content of selected bioactive components in fermented food products from two production systems are presented in Table 1. 

The lactic acid bacteria number in the tested samples varied. Pickled carrot juice contained the least microorganisms, while sauerkraut contained the most (10^4^ and 10.7 × 10^8^ CFU per mL or g, respectively). In both cases, significantly more LAB were isolated from organic products than from conventional ones. A similar tendency was reported with organic yogurt and kefir, which contained significantly more lactic acid bacteria than their conventional counterparts, with the difference being most evident in yogurt, reaching three orders of magnitude. Conventional pickled beet juice and conventional pickled cucumbers are the only products tested that contained significantly more LAB (*p* < 0.05) compared to organic production. 

Among all analyzed plant products, regardless of the production system, a much higher concentration of vitamin C was found in juices than in vegetables. Sauerkraut and pickled cucumbers contained several or several dozen times less of this compound. Organic vegetables contained significantly more ascorbic acid than their conventional counterparts (*p* < 0.05), while in juices, the concentration of vitamin C in conventional and organic products did not differ. 

The content of β-carotene was determined in pickled beetroot and carrot juice from different production systems, and relatively similar concentrations for both production systems were determined in the latter product. On the other hand, in organic pickled beetroot juice, the concentration of ß-carotene was five-fold lower than in its conventional counterpart.

The calcium content in the fermented milk products ranged from 127.52 to 165.75 mg·100 g^−1^; it was only significantly higher (*p* < 0.05) in organic yogurt compared to its conventional version. As for kefir and buttermilk, the production system did not significantly affect the concentration of Ca. 

A total of 70 isolates of lactic acid bacteria isolated from fermented products (five for each product type) were screened for bacteriocin production. Each of them inhibited the growth of at least two indicator bacteria. The mean sizes of the zones of growth inhibition are presented in Table 2. The analysis of bacteriocinogenic properties of LAB indicates a more comprehensive antagonistic potential of LAB isolated from fermented dairy products (Table 2). They inhibited the development of all tested pathogens, both gram-positive and gram-negative, and the average size of the growth inhibition zone, regardless of the production system, ranged from 8.15 to 11.67 mm. On the other hand, isolates derived from food of plant origin were more effective against gram-positive bacteria, i.e., *L. monocytogenes* and *S. aureus*. The mean sizes of the inhibition zones of all pathogens caused by LAB bacteriocins isolated from plant products reached 6.25 to 7.17 mm. Significantly larger zones (*p* < 0.05) of growth inhibition of all pathogens were induced by isolates derived from organic products (pickled beet juice, dairy products). The only exceptions were LAB isolated from conventional pickled cucumbers, which were more effective in inhibiting the growth of *L. monocytogenes*, and isolates from conventional sauerkraut, which impeded the growth of *L. monocytogenes* and *S. aureus* compared to organic products. In the case of LAB derived from pickled carrot juice, no significant differences were found in the bacteriocinogenic activity between isolates from conventional and organic products.

## 3. Discussion

It is believed that modern methods of food production, aimed at continuous increase in their efficiency with a simultaneous reduction of costs, do not ensure the production of foods of sufficiently high health quality. These products often contain an excess of nitrates, pesticide residues, and heavy metals, and thus cause allergies, reduce the body’s resistance, and contribute to the development of diseases across civilizations. Therefore, with the constantly growing ecological and nutritional awareness of society, an increasing number of consumers are looking for organic food from certified organic farms. Such food, apart from its nutritional function, can also contribute to maintaining good health and constitute an important element of health issue prevention in society. Raw materials (vegetables, fruit, milk) obtained by both conventional and organic methods can be enriched naturally with bioactive compounds by subjecting them to the process of lactic fermentation. These compounds are metabolites of food-fermenting lactic acid bacteria, which guarantee the sensory properties, shelf life, and safety of most fermented foods. Many of them are antagonistic to food-borne pathogens and spoilage microorganisms (e.g., organic acids, diacetyl, carbon dioxide, lactoperoxidase system, bacteriocins). Lactic acid bacteria themselves are also a very important bioactive component of the product due to their potential probiotic properties. As summarized by Rezac et al. [15], the LAB number in fermented food accounts for 10^5^–10^9^ CFU per mL or gram, with dairy products containing the highest amount. No such tendency was observed in the present study. The LAB number in products of plant origin ranged from 1.0 × 10^4^ to 10.7 × 10^8^ CFU·mL^−1^ or g^−1^, whereas in dairy products, from 33 × 10^4^ to 75.6 × 10^7^ CFU per g. Regarding the production system, there were significantly more LAB in organic yogurt and kefir than in their conventional counterparts. Likewise, Hanus et al. [16] identified almost twice as many lactic acid bacteria in organic milk samples than in milk from conventional farms (138 × 10^7^ and 75 × 10^7^ CFU·mL^−1^, respectively). They also reported that organic milk can be a slightly better environment for yogurt fermentation due to its acidity. The results of the present research do not give a clear answer which of the production systems favor the development of LAB in fermented food of plant origin. Significantly more LAB were found in organic pickled carrot juice and sauerkraut than in their conventional counterparts, while conventional pickled beet juice and conventional pickled cucumbers contained significantly more LAB compared to organic production. 

Fruits, vegetables, milk, and their fermented products are essential components of a healthy diet. The content and availability of bioactive components in fermented foods may differ from that in raw materials. For example, a low pH slows down the enzymatic processes, which means that during fermentation, a significant amount of ascorbic acid is retained in vegetables [17]. Moreover, LAF (lactic acid fermentation) allows for β-carotene retention at the level of 93.94%, and even enriches the products in carotenoids [18,19]. Lactic acid fermentation of tomato pulp supplemented with different lactic acid bacteria contributed to the increase in the level of total carotenoids by 33.6–41.1%, lycopene by 24.8–50%, and the content of β-carotene by 69% [20]. With fermented milk drinks, an increase in the content and bioavailability of minerals, especially calcium, phosphorus, potassium, zinc, and magnesium, resulting from the activity of lactic acid bacteria was reported [21,22]. Therefore, it is worth including fermented products, preferably from organic production, in the diet. As previously reported, organic plant raw materials contain fewer chemical residues, but more dry matter, sugars, mineral components, and vitamin C [23]. These findings were confirmed by Kazimierczak et al. [24] and Sikora et al. [25] by analyzing fresh and fermented beetroots grown in a conventional and organic system. Both fresh and fermented organic products contained significantly more vitamin C than the fresh conventional beetroots; however, other studies did not confirm these differences and a higher content of vitamin C in organic products [3,26,27]. The present research also did not give a clear answer on which of the production systems allows for obtaining a higher concentration of this vitamin. It was found that while organic pickled vegetables (cucumbers and cabbage) contained significantly more ascorbic acid than conventional ones, the concentration of vitamin C did not differ significantly in the juices from different production systems.

Another bioactive substance analyzed in this research was β-carotene found in orange, yellow fruit, and green leafy vegetables. Several authors compared the content of carotenoids in organic and conventional plant products and obtained different results. Some of them found a higher concentration of these substances in organic vegetables, e.g., carrots, tomatoes, and cabbage. El-Bassel and El-Gazzar [28] concluded that the organic vegetables have higher β-carotene content than the non-organic ones, ranging from 18.5% for carrots to 39% for tomatoes. Sikora et al. [29], on the other hand, reported organic carrots as significantly more abundant in vitamins, carotenoids, and phenolic acids in comparison to the conventional ones. In the present study, a significantly higher content of β-carotene was found in conventional pickled beetroot juice than in organic juice (1.0656 and 0.2407 mg per 100 mL, respectively). Similar results were reported by Pavlović et al. [30] regarding the content of β-carotene in fresh and processed beet and carrot, and their products. Other researchers also indicate a higher content of bioactive substances, including β-carotene, in vegetables from conventional crops [31,32]. On the other hand, in carrot juice, no significant differences were found in the content of β-carotene depending on the production system (17.098 mg–conv and 16.7481 mg–org per 100 mL).

Calcium was another nutrient analyzed in this study. A good source of calcium in the diet is cow’s milk and its derivatives. The content of minerals in milk depends mainly on their level in the feed and the animal feeding system, which is different between organic and conventional farms. According to Toledo et al. [33], organic milk may contain less minerals compared to that obtained on conventional farms. Organic farming limits the application of inorganic supplements, therefore, mineral deficiencies may occur because of the low availability of some trace elements. Koperska et al. [34] reported that milk from organic farms had a significantly (*p* ≤ 0.05) lower content of Ca, Mg, Zn, Mn, and Cu. Other researchers, on the other hand, indicate a higher content in organic milk of Ca, K, P, and Mo, but lower content of Cu, Fe, Mn, Zn, and Al than in conventional milk [35]. Other groups of researchers found no significant differences between organic and conventional milk [36]. In the present study, the calcium content in fermented milk products ranged from 127.52 to 165.75 mg·100g^−1^, regardless of the production system. These data are in agreement with Kłobukowski et al. [37], who reported that the content of this microelement in 100 g of kefir, yogurt, or buttermilk was 103–170 mg. The results of the present research did not confirm a correlation between the level of calcium content and the production system of fermented milk drinks. Only in organic yogurt was there significantly more calcium than in the conventional product (165.75 and 153.80 mg·100 mL^−1^, respectively). As for buttermilk and kefir, the content of Ca was similar, regardless of the production system used.

Due to the wide spectrum of antibacterial substances produced by lactic acid bacteria–such as organic acids, hydrogen peroxide, or bacteriocins–they have a great potential to fight unwanted microorganisms in food products, including pathogens. Bacteriocins differ in the spectrum of antimicrobial activity. Some of them are antagonistic to species closely related to the producers, whereas others have a broad antibacterial spectrum [38,39,40]. Our findings are in agreement with those reports. Lactic acid bacteria were isolated from two main source-fermented plant products and fermented dairy products, which came from different production systems (org and conv), and the effects of bacteriocins produced by them against both gram-positive (*Listeria monocytogenes*, *Staphylococcus aureus*) and gram-negative bacteria (*Escherichia coli* and *Salmonella* Senftenberg) were tested. It was found that, regardless of the production system, LAB isolated from dairy products had a more comprehensive antagonistic potential against all pathogens tested, while isolates derived from pickled juices and vegetables were more effective against gram-positive bacteria. This situation was perhaps caused by the supplementation of fermented dairy products with starter cultures of lactic acid bacteria (including strains with a broad spectrum of antimicrobial activity), the presence of which is declared by the producers on the labels. It is possible that the fermented plant products tested in this study were produced as a result of the enzymatic activity of the natural microbiota of raw materials, which does not show as strong bacteriocinogenic properties as starter cultures in dairy fermented food. As for the analysis of the size of the zones of pathogen growth inhibition, it can be concluded that isolates derived from organic products showed stronger antagonism against all pathogens. *L. monocytogenes* and *S. aureus* were more sensitive to the action of bacteriocins, while *Salmonella* Senftenberg and *Escherichia coli* were more resistant. These observations are consistent with the reports by Sezer and Güven [41], who found that *Leuconostoc mesenteroides* ssp. *mesenteroides* isolated from butter and *Lactobacillus brevis* isolated from butter, cream, and kefir showed strong antimicrobial activity against *L. monocytogenes* and *S. aureus* but not against the strains of *E. coli*, *Y. enterocolitica*, and other gram-negative bacteria. On the other hand, Choi et al. [42] reported a full inhibition of foodborne pathogenic bacteria, i.e., *E. coli* O157:H7, *Salmonella* Enteritidis, *Salmonella* Typhimurium, and *S. aureus* by lactic acid bacteria derived from a plant-based fermented food (kimchi). Jiang et al. [43] have isolated from Yunnan traditional fermented yogurt a strain of *L. paracasei* LS-6, which produces a novel bacteriocin–named LSX01–of broad-spectrum antibacterial activity. For example, LSX01 exhibited the highest antibacterial activity against various strains of *S. aureus* (an inhibitory zone of about 25 mm). It is also effective against gram-negative bacteria, such as *Escherichia coli* ATCC 3521 (23.69 ± 0.15 mm) and *Salmonella* Enteritidis CICC21482 (18.36 ± 0.22 mm). Compared to the results of the present study, the indicated zones of growth inhibition are much larger, especially with gram-negative bacteria. However, it seems that regardless of the type of fermented food and its production system, it is a good source of lactic acid bacteria, which are potentially probiotic.

Research results do not clearly indicate which production system–conventional or organic–provides higher levels of bioactive substances in fermented food. However, it should be emphasized that the LAB number and their bacteriocinogenic potential was higher in most organic products. Organic vegetables (but not organic juices) contained significantly more vitamin C, and organic yogurt was richer in calcium compared to their conventional counterparts. On the other hand, relatively similar concentrations of ß-carotene were found in both organic and conventional carrot juice, while in organic pickled beet juice, there was five-fold less of this compound than in conventional one. 

## 4. Materials and Methods

The research material included organic and conventional fermented food of plant and animal origin. Its list and characteristics are given in Table 3. The choice of products was driven by growing consumer interest in fermented foods, which are perceived as natural and health-promoting, especially in terms of their positive effects on the human microbiome. In addition, plant-based fermented beverages can provide a readily available alternative to fermented dairy products for people with lactose intolerance or a milk protein allergy.

### 4.1. Microbiological Testing

#### 4.1.1. Isolation of Lactic Acid Bacteria from the Tested Food Samples 

The microbiological analysis of selected plant and dairy products was carried out using the method of tenfold dilution of food samples in 0.85% NaCl and surface seeding on the MRS medium (Merck, 69964) [44]. The pickled vegetables were ground, then 10 g of the sample was transferred to 90 mL of saline, while with food of liquid consistency, 10-mL samples were taken and transferred to 90-mL flasks with 0.85% NaCl. The samples were shaken for 30 min, diluted in the range of 10^−1^–10^−7^, and 0.1 mL of the samples were then transferred to the surface of the medium. The material was thoroughly spread with a brush. Three replicates were prepared for each dilution. The cultures were incubated at 30 °C for 48 h, after which the colonies with a morphology typical of lactic acid bacteria were counted. To confirm their taxonomic affiliation, microscopic preparations were prepared and stained with the Gram method, and a test for the production of catalase was carried out. Five isolates from each fermented product, gram-positive, catalase-negative bacilli, and streptococci, were selected for further research. A total of 70 pure cultures were obtained and stored on MRS slants at 4 °C. 

#### 4.1.2. Determination of Bacteriocinogenic Properties of Lactic Bacteria against Pathogens

The experiment was conducted according to Dopazo et al. [45], with modification. The microorganisms against which the effectiveness of the bacteriocins was tested were *Escherichia coli*, *Salmonella* Senftenberg W775, *Listeria monocytogenes*, and *Staphylococcus aureus*. Lactic bacteria were inoculated on MRS liquid medium (Merck, 10661) and incubated at 30 °C for 24 h, after which each suspension was adjusted to an optical density of 0.5 McFarland. A total of 5 µL of each suspension was transferred to a solid MRS medium and incubated at 37 °C under anaerobic conditions (AnaeroGen, Oxoid) for 24 h. Next, the cultures were treated with chloroform for 20 min to kill the microorganisms and avoid the production of lactic acid and H_2_O_2_ in the further steps of the experiment. The excess reagent was evaporated over the following 20 min. In the next stages of the experiment, the method of two-layer tiles was used. To the liquefied solid TSA media (Merck, 22091), 0.1 mL of an 18-h culture of the tested pathogens was added, and this mixture was transferred to the surface of a medium containing bacteriocins secreted by lactic bacteria. As a control, plates without LAB in the lower layer were employed. The cultures were incubated at 37 °C for 24 h under aerobic conditions. The antagonistic properties of lactic bacteria were assessed by measuring the diameter of the inhibition zone of pathogen growth.

### 4.2. Evaluation of the Content of Bioactive Compounds

#### 4.2.1. Determination of Vitamin C Content

The content of ascorbic acid was determined by titration in an acid medium with a standard solution of 2,6-dibromophenolindophenol dye until a pink color appeared. The sample preparation consisted of grinding 2 g of plant material with 5 mL of 3% metaphosphoric acid in 8% acetic acid, or adding 2 mL of liquid sample to the above-mentioned mixtures of acids. The mixture was made up to 10 mL with the acid, and 5 mL of the liquid was taken and titrated with the standard dye solution until the color remained pink for 30 s [46].

#### 4.2.2. Determination of β-Carotene Content

The analysis was performed for juices from pickled vegetables. About 10–20 g of the liquid products were transferred to a separating funnel by rinsing with 20 mL of acetone, then 15 mL of hexane was added, and the contents were extracted by shaking. β-carotene penetrated the upper hexane layer. After phase separation, the bottom acetone–water layer was drained into a conical flask for further extraction with hexane. A second extraction with 10 mL of hexane was done. The collected hexane layers were washed with 25 mL of distilled water (no shaking) and the lower aqueous layer was removed after separation. After 10 min, the residual water was separated, the obtained extract was transferred to a measuring cylinder, and its volume was read (V0). The absorbance was measured on a Shimadzu UV-1800 UV-VIS spectrophotometer at a wavelength of 450 nm, in 10 mm thick cuvettes, against a mixture of hexane and acetone [47].

#### 4.2.3. Determination of Calcium Content

Total calcium determination in yogurt, kefir, and buttermilk was carried out according to a modified version of the procedure of Minczewski and Marczenko [48]. Briefly, a 0.5 g sample of the product was diluted with distilled water (200 mL) and homogenized using a magnetic stirrer. After a dozen minutes, it was made alkaline with 2 mL of 8 M NaOH solution (to pH 12–13) to precipitate magnesium ions. Next, 0.6 mL of 0.002M calcein solution was added and the sample was titrated with disodium edetate (EDTA). The image of the reaction vessel was captured each time an aliquot of titrant was added using a Logitech webcam. The ChemiON software was used to control the measurement and data acquisition. The titration curves were obtained by plotting the value of a specific color component (R-G-B) read from the recorded photos as a function of the volume of added titrant. Based on the curves, the titration endpoint was determined as the volume of titrant used to reduce the green color component or increase the red component. The calcium content in the tested products was calculated from the formula:

$$m_{\mathrm{Ca}}=\frac{C_{\mathrm{EDTA}}\times V_{\mathrm{EDTA}}\times M_{\mathrm{Ca}}\times 100}{m}$$

where: m_Ca_—the mass of calcium in 100 g of the product [mg·100g^−1^], C_EDTA_—titrant concentration [mol·dm^−3^], V_EDTA_—volume of the titrant at the endpoint of the titration [mL], M_Ca_—molar mass of calcium [g·mol^−1^], m—weight of the analyte [g]. 

### 4.3. Statistical Analysis

The results are the mean of three replications from each product analysed. The results were statistically analyzed using analysis of variance (one-way and two-way ANOVA). To show statistically significant differences between selected features (LAB number, content of vitamin C, carotene, and Ca) of the tested products grown in organic and conventional system, one-way ANOVA analysis was performed. The differences between the ability to produce bacteriocins by LAB strains isolated from vegetables grown under organic and conventional systems were investigated using two-way ANOVA, where the first factor was the production systems (A) and the second was microorganism species (B). The significance of differences between the values was determined by Tukey’s test, at *p* ≤ 0.05. The statistical analysis of the results was performed using the Statistica.PL 12 [49] software package.

## 5. Conclusions

The demand for organic food shows a constant upward trend. It is the result of the growing interest of consumers in a healthy lifestyle, the dissemination of environmentally friendly production methods, and the demand for good food quality. The use of the lactic acid fermentation process for food preservation gives additional benefits that allow for the development of the desired sensory and health properties of the product. However, the quality of organic food is not always better than conventional food. This can be due to many factors (e.g., production technology, the chemical composition of the raw material, or storage conditions). The results of this study show that organic vegetables (sauerkraut and cucumbers) had more vitamin C, while organic pickled beet juice contained five times less ß-carotene than its conventional counterpart. Organic yogurt contained a higher Ca content than the conventional one. The LAB number in organic pickled carrot juice, sauerkraut, and organic yogurt and kefir was higher, whereas in organic pickled beetroot juice and pickled cucumbers it was lower compared to conventional production. LAB isolated from most organic products had a higher antagonistic activity resulting from the production of bacteriocins. So far, no unambiguous results have been found indicating the superiority of organic food over conventionally produced food; therefore, much more research is needed.

## Figures and Tables

**Table 1:** Results of qualitative and quantitative analysis of fermented food products from two production systems.

Product	Production System	LAB Number (cfu/1 g or cfu/1 mL)	Vitamin C Content (mg/100 g or mg/100 mL)	β-Caroten Content (mg/100 mL)	Ca Content (mg/100 g)
pickled beet juice	org	2.03 × 10^6 b^	28.56	0.2407 ^b^	n.d.
	conv	10.1 × 10^6 a^	31.25	1.0656 ^a^	n.d.
		LSD = 2.134	n.s.	LSD = 0.134	
pickled carrot juice	org	2.00 × 10^4 a^	25.28	16.7481	n.d.
	conv	1.00 × 10^4 b^	32.05	17.0980	n.d.
		LSD = 0.863	n.s.	n.s.	
pickled cucumbers	org	1.00 × 10^5 b^	1.7822 ^a^	n.d.	n.d.
	conv	30.00 × 10^5 a^	0.8650 ^b^	n.d.	n.d.
		LSD = 12.828	LSD = 0.331		
sauerkraut	org	10.70 × 10^8 a^	5.3678 ^a^	n.d.	n.d.
	conv	0.12 × 10^8 b^	3.5538 ^b^	n.d.	n.d.
		LSD = 12.034	LSD = 0.685		
yogurt	org	75.6 × 10^7 a^	n.d.	n.d.	165.75 ^a^
	conv	0.033 × 10^7 b^	n.d.	n.d.	153.80 ^b^
		LSD = 12.745			LSD = 7.473
kefir	org	61.6 × 10^6 a^	n.d.	n.d.	129.40
	conv	45.6 × 10^6 b^	n.d.	n.d.	127.52
		LSD = 8.743			n.s.
buttermilk	org	4.40 × 10^7^	n.d.	n.d.	137.77
	conv	4.00 × 10^7^	n.d.	n.d.	135.06
		n.s.			n.s.

org—organic production system; conv—conventional production system; LSD—Least Significant Difference; ^a–b^—letters in columns indicate significant differences between values for individual groups of products from analyzed production systems at *p* < 0.05 (One-Way ANOVA); n.d.—not determined; n. s.—not significant.

**Table 2:** Size of the inhibition zones of pathogen growth [mm] induced by bacteriocins of lactic acid bacteria isolated from fermented food.

Product	Production System	Pathogen				Mean A
		*Escherichia coli*	*Salmonella* Senftenberg	*Listeria monocytogenes*	*Staphylococcus aureus*	
pickled beet juice	org	1.5	2.1	15.4	11.2	7.55 ^a^
	conv	1.7	1.9	13.9	9.8	6.82 ^b^
	Mean B	1.60 ^c^	2.00 ^c^	14.65 ^a^	10.5 ^b^	
LSD for: Factor A = 0.685, Factor B = 1.346; Interaction A/B = n.s. B/A = n.s.						
pickled carrot juice	org	0.0	0.0	8.7	7.8	4.12 ^a^
	conv	0.0	0.0	9.2	8.4	4.40 ^a^
	Mean B	0.00	0.00	8.95 ^a^	8.10 ^b^	
LSD for: Factor A = n.s., Factor B = 0.65; Interaction A/B = n.s. B/A = n.s.						
pickled cucumbers	org	2.0	1.	10.5	8.9	5.75 ^a^
	conv	1.8	1.0	12.3	9.5	6.15 ^a^
	Mean B	1.90 ^c^	1.30 ^c^	11.40 ^a^	9.17 ^b^	
LSD for: Factor A = n.s., Factor B = 0.842; Interaction A/B = 0.857 B/A = 1.190						
sauerkraut	org	0.6	0.7	7.5	6.2	3.75 ^b^
	conv	0.9	1.0	9.1	7.1	4.53 ^a^
	Mean B	0.75 ^c^	0.85 ^c^	8.30 ^a^	6.65 ^b^	
LSD for: Factor A = 0.355., Factor B = 0.698; Interaction A/B = 0.711 B/A = 0.987						
yogurt	org	7.4	7.1	20.1	17.2	12.95 ^a^
	conv	6.1	6.6	19.4	15.6	11.92 ^b^
	Mean B	6.75 ^c^	6.85 ^c^	19.73 ^a^	16.40 ^b^	
LSD for: Factor A = 0.497, Factor B = 0.976; Interaction A/B = n.s. B/A = n.s.						
kefir	org	4.5	4.6	17.6	14.3	10.25 ^b^
	conv	5.1	5.2	18.5	15.1	10.97 ^a^
	Mean B	4.8 ^c^	4.9 ^c^	18.05 ^a^	14.7 ^b^	
LSD for: Factor A = 0.382, Factor B = 0.751; Interaction A/B = n.s. B/A = n.s.						
buttermilk	org	5.3	4.2	18.6	16.8	11.22 ^a^
	conv	4.9	3.6	18.5	16.1	10.77 ^b^
	Mean B	5.10 ^c^	3.90 ^d^	18.55 ^a^	16.45 ^b^	
LSD for: Factor A = 0.351, Factor B = 0.689; Interaction A/B = n.s. B/A = n.s.						

Factor A levels: production system (organic, conventional); Factor B levels (bacteria species); LSD—Least Significant Difference, ^a–d^—letters indicate significant differences between mean values in columns (A factor) and rows (B factor) at *p* < 0.05. (Two-Way ANOVA); n.s.—not significant.

**Table 3:** Characteristics of fermented food products used in the study.

Product	Production System	Components	Nutritional Value in 100 g (100 mL) of the Product
Pickled cucumbers	org	cucumber, dill, horseradish, garlic, spring brine	energy value-fat-0.1 g, including saturated fatty acids-0.0 g; carbohydrates-1.9 g, including sugars-0.00 g; protein-0.6 g; salt-2.1 g
	conv	cucumbers, table salt, dill, horseradish, garlic, spices	energy value-50 kcal/12 kJ; fat-0 g, including saturated fatty acids-0 g; carbohydrates-1.9 g, including sugars-0 g; protein-1 g; salt-1 g
Sauerkraut	org	white cabbage, carrots, non-iodized rock salt	energy value-71 kJ/17 kcal; fat-0.2 g, including saturated fatty acids-0.0 g; carbohydrates-2.3 g, including sugars-2.0 g; protein-0.9 g; salt-0.8 g
	conv	white cabbage, carrots, table salt	data not available
Pickled beet juice	org	beetroot, dill, horseradish, garlic, natural spring brine	energy value-76 kJ/18 kcal; fat-0.04 g, including saturated fatty acids-0.0 g; carbohydrates-3.6 g, including sugars-0.15 g; protein-0.9 g; salt-1.7 g
	conv	naturally pickled red beet extract, garlic, salt, spices, citric acid	energy value-50 kJ/12 kcal; fat-0.2 g, including saturated fatty acids-0.2 g; carbohydrates < 0.3 g, including sugars < 0.1 g; protein < 0.3 g; salt-1.85 g
Pickled carrot juice	org	organic pickled carrot juice	energy value-155 kJ/37 kcal; fat-0.1 g, including saturated fatty acids-0.08 g; carbohydrates-8.0 g, including sugars-7.7 g; protein-0.5 g; salt-0.15 g
	conv	pickled carrot juice (80%), apples (20%), mixed spices (salt, garlic, horseradish, allspice, bay leaf)	energy value-65 kJ/15 kcal; fat-0.00 g, including saturated fatty acids-0.00 g; carbohydrates-2.99 g, including sugars-2.45 g; protein-0.43 g; salt-0.93 g
Yogurt	org	whole milk, skimmed milk powder, lactic acid bacteria (*Streptococcus thermophilus, Lactobacillus bulgaricus*)	energy value-316 kJ/75 kcal; fat-3.8 g, including saturated fatty acids-2.4 g; carbohydrates-4.7 g, including sugars-4.7 g; protein-5.0 g; salt-0.15 g
	conv	milk, milk proteins, lactic acid bacteria	energy value-280 kJ/67 kcal; fat-3.1 g, including saturated fatty acids-2.1 g; carbohydrates-4.0 g, including sugars-4.0 g; protein-4.8 g; salt-0.17 g
Kefir	org	organic milk, lactic acid bacteria, and kefir yeast	energy value-208 kJ/50 kcal; fat-2.0 g, including saturated fatty acids-1.3 g; carbohydrates-4.7 g, including sugars-4.7 g; protein-3.2 g; salt-0.10 g
	conv	skim milk, cream (from milk), skim milk powder, lactic acid bacteria, kefir yeast	energy value-215 kJ/51 kcal; fat-1.5 g, including saturated fatty acids-0.9 g; carbohydrates-6.7 g, including sugars-6.7 g; protein-2.7 g; salt-0.10 g
Buttermilk	org	natural buttermilk, lactic acid bacteria	energy value-135 kJ/32 kcal; fat-0.7 g, including saturated fatty acids-0.4 g; carbohydrates-3.6 g, including sugars-3.6 g; protein-2.6 g; salt-0.1 g
	conv	natural pasteurized buttermilk, pasteurized milk, lactic acid bacteria	energy value-190 kJ/45 kcal; fat-1.5 g, including saturated fatty acids-0.9 g; carbohydrates-4.5 g, including sugars-4.5 g; protein-3.4 g; salt-0.04 g

org—organic production system; conv—conventional production system.

## Data Availability

Not Applicable.

## Sample Availability

Samples of the compounds are not available from the authors.
